# Transcriptomes of *Ralstonia solanacearum* during Root Colonization of *Solanum commersonii*

**DOI:** 10.3389/fpls.2017.00370

**Published:** 2017-03-20

**Authors:** Marina Puigvert, Rodrigo Guarischi-Sousa, Paola Zuluaga, Núria S. Coll, Alberto P. Macho, João C. Setubal, Marc Valls

**Affiliations:** ^1^Department of Genetics, University of BarcelonaBarcelona, Spain; ^2^Centre for Research in Agricultural Genomics CSIC-IRTA, Autonomous University of BarcelonaBellaterra, Spain; ^3^Department of Biochemistry, University of São PauloSão Paulo, Brazil; ^4^Shanghai Center for Plant Stress Biology, CAS Center for Excellence in Molecular Plant Sciences, Shanghai Institutes of Biological Sciences, Chinese Academy of Sciences (CAS)Shanghai, China

**Keywords:** *Ralstonia solanacearum*, bacterial wilt, *Solanum commersonii*, RNA sequencing, transcriptomics, disease resistance, potato brown rot

## Abstract

Bacterial wilt of potatoes—also called brown rot—is a devastating disease caused by the vascular pathogen *Ralstonia solanacearum* that leads to significant yield loss. As in other plant-pathogen interactions, the first contacts established between the bacterium and the plant largely condition the disease outcome. Here, we studied the transcriptome of *R. solanacearum* UY031 early after infection in two accessions of the wild potato *Solanum commersonii* showing contrasting resistance to bacterial wilt. Total RNAs obtained from asymptomatic infected roots were deep sequenced and for 4,609 out of the 4,778 annotated genes in strain UY031 were recovered. Only 2 genes were differentially-expressed between the resistant and the susceptible plant accessions, suggesting that the bacterial component plays a minor role in the establishment of disease. On the contrary, 422 genes were differentially expressed (DE) *in planta* compared to growth on a synthetic rich medium. Only 73 of these genes had been previously identified as DE in a transcriptome of *R. solanacearum* extracted from infected tomato xylem vessels. Virulence determinants such as the Type Three Secretion System (T3SS) and its effector proteins, motility structures, and reactive oxygen species (ROS) detoxifying enzymes were induced during infection of *S. commersonii*. On the contrary, metabolic activities were mostly repressed during early root colonization, with the notable exception of nitrogen metabolism, sulfate reduction and phosphate uptake. Several of the *R. solanacearum* genes identified as significantly up-regulated during infection had not been previously described as virulence factors. This is the first report describing the *R. solanacearum* transcriptome directly obtained from infected tissue and also the first to analyze bacterial gene expression in the roots, where plant infection takes place. We also demonstrate that the bacterial transcriptome *in planta* can be studied when pathogen numbers are low by sequencing transcripts from infected tissue avoiding prokaryotic RNA enrichment.

## Introduction

Changes in pathogen gene expression control the switch from a commensal to a parasitic relationship with the host, which may subvert the host metabolism or development to the pathogen's benefit (Stes et al., 2011). However, there is still limited information concerning how this is controlled. Understanding how these trophic relationships initiate and persist in the host requires deciphering the functional adaptations at the transcriptomic level. Pioneer studies of the expression profiles of bacterial animal pathogens in infected tissues showed that the genes induced more strongly contributed to bacterial virulence and/or survival in the host (reviewed in La et al., 2008).

*Ralstonia solanacearum* is the causal agent of the destructive bacterial wilt disease in tropical and subtropical crops, including tomato, tobacco, banana, peanut, and eggplant (Hayward, 1991; Peeters et al., 2013). The disease in potato is also called brown rot and is endemic in the Andean region, where potato is a staple food, causing an important impact on food production and the economy (Priou, 2004; Coll and Valls, 2013). Disease control of bacterial wilt is very challenging, because of the bacterium aggressiveness, its persistence in the field and the lack of resistant commercial varieties in any of its hosts. Potato breeding programs have used wild species related to *Solanum tuberosum*, such as *Solanum commersonii*, as sources of resistance against bacterial wilt (Kim-Lee et al., 2005; Siri et al., 2009).

As in most Gram-negative animal and plant pathogens, the major pathogenicity determinant in *R. solanacearum* is the type three secretion system (T3SS) (Boucher et al., 1987). This system injects bacterial proteins called effectors directly into the eukaryotic host cells to manipulate the host defenses and establish disease (Buttner, 2016; Popa et al., 2016a). Amongst other factors that contribute to *R. solanacearum* virulence are motility—either caused by flagella or type IV pili- and the reactive oxygen species (ROS)- detoxifying enzymes (Meng, 2013).

*In vitro* studies using microarrays allowed the study of *R. solanacearum* virulence gene expression and the discovery of novel regulatory networks (Occhialini et al., 2005; Valls et al., 2006). However, the first studies on gene expression *in planta* using quantitative reporters indicated that *R. solanacearum* virulence genes showed unexpected expression patterns (Monteiro et al., 2012). Contrary to what was believed based on *in vitro* studies, it was demonstrated that the genes encoding the T3SS genes and its associated effectors were transcribed *in planta* at late stages of infection (Monteiro et al., 2012). These findings were later confirmed in transcriptomic studies with *R. solanacearum* extracted from infected tomato and banana plants (Jacobs et al., 2012; Ailloud et al., 2016). However, these studies *in planta* could only be performed from heavily colonized plants, as limited pathogen biomass has hindered until recently the investigation of gene expression at the early stages of the interaction, when plants are still asymptomatic.

In a previous work, we demonstrated that rRNA-depleted RNAs obtained from infected roots could be used to determine the transcriptomic responses of *S. commersonnii* plants resistant or susceptible to bacterial wilt through RNA sequencing (Zuluaga et al., 2015). Here, we have used these sequences to extract *R. solanacearum* UY031 transcripts *in silico* and have compared them to the bacterial transcriptomes obtained in synthetic media to investigate the pathogen RNAs expressed during early infection. Our results reveal differential expression of a number of known and putative transcriptional regulators and virulence factors during early plant colonization, providing insight into their role in infection.

## Materials and methods

### Bacterial strains, plant accessions, and growth conditions

The *R. solanacearum* isolate UY031, phylotype IIB, sequevar 1, originally isolated from potato (Siri et al., 2011), carrying the LUX-operon under the *psbA* promoter (Monteiro et al., 2012) was used for all experiments. Bacteria were routinely grown in rich B medium as described (Monteiro et al., 2012).

*S. commersonnii* accessions F97 (susceptible to bacterial wilt) and F118 (moderately resistant) obtained from a segregating population were used in this work and propagated *in vitro* as described (Zuluaga et al., 2015).

### Sample preparation

As a control condition, bacteria were grown for 2 days on rich solid medium without tetrazolium chloride or antibiotics at the appropriate dilution to obtain separate colonies. Bacteria were recovered from plates and mixed with 5% of an ice-cold transcription stop solution [5% (vol/vol) water-saturated phenol in ethanol]. Cells were centrifuged at 4°C for 2 min at maximum speed and the bacterial pellet was immediately frozen in liquid nitrogen.

For plant RNA samples, *S. commersonii* F97 and F118 roots were inoculated as described in Zuluaga et al. (2015). Briefly, plant roots from 2-week old plants grown in soil were injured with a 1 ml pipette tip and inoculated by soil drenching with a bacterial solution at 10^7^ colony forming units (cfu)/ml. Control plants were mock-inoculated with water. After inoculation, plants were kept in a growth chamber at 28°C in long-day conditions. Luminescence quantification was used to select plants with comparable infection levels in the susceptible and the resistant accessions, corresponding to approximately 10^5^ colony forming units per g of tissue (Cruz et al., 2014).

### RNA extraction, sequencing, and library preparation

Total RNA from bacterial cultures was extracted using the SV Total RNA Isolation System kit (Promega) following the manufacturer's instructions for Gram-negative Bacteria. Infected plant RNA extractions were carried out as described (Cruz et al., 2014). RNA concentration and quality was measured using the Agilent 2100 Bioanalyzer. For rRNA depletion, 2.5 μg of RNA were treated with the Ribo-zero^(™)^ magnetic kit for bacteria (Epicenter). Three biological replicates per condition were subjected to sequencing on an Illumina-Solexa Genome Analyzer II apparatus in the Shanghai PSC Genomics facility using multiplexing and kits specially adapted to obtain 100 bp paired-end reads in stranded libraries. Raw sequencing data is available in the Sequence Read Archive under the accession code SRP096020.

### Read mapping, quantification, and differential gene expression analysis

FASTQC was used to evaluate the quality of the RNA-seq raw data. *R. solanacearum* reads were identified from total infected root sequences using Bowtie2 (version 2.2.6; Langmead and Salzberg, 2012) as described in the results section. The completely sequenced genome of strain UY031 (Guarischi-Sousa et al., 2016) was used as reference. For identification of *R. solanacearum* reads, the Burrows-Wheeler Alignment (BWA) tool was initially used. However, a high number of reads from mock-inoculated control samples mapped to the bacterial genome (Table 1). Visual evaluation of these mapped reads using the Integrative Genomics Viewer (IGV) tool (Robinson et al., 2011; Thorvaldsdottir et al., 2013) showed that most contained mismatches to the *R. solanacearum* genome sequence, indicating that they likely belonged to contaminating bacteria. BWA was thus assayed with more stringent parameters (-B 20-O 30-E 5-U 85), to increase penalties for mismatches, gap openings, gap extension, and unpaired read pairs, resulting in a reduction of only half of the reads mapping to the genome. Finally, Bowtie2 was assayed, once more using stringent parameters to penalize mismatches and gaps (–mp 30–rdg 25,15–rfg 25,15). In this case, mapped reads levels in mock-inoculated plants could be considered background compared to the high read numbers from inoculated samples, thus, Bowtie2 was finally used in all samples analyzed, including RNA-seq reads coming from *in vitro* grown bacteria (Table 1). Alignments were summarized by genes on counting tables using HTSeq-count (version 0.6.1 p1; Anders et al., 2015) and NCBI's reference annotation (genome features were extracted from NCBI's RefSeq sequences NZ_CP012687.1 and NZ_CP012688.1); alignments with quality lower than 10 were discarded. Differential expression (DE) analysis was carried out with the DESeq2 (version 1.12.3; Love et al., 2014) package in R (version 3.3.2). Benjamini–Hochberg procedure was used for multiple testing corrections. Genes with log_2_(fold-change) > 0.5 and *q* < 0.01 were considered as differentially expressed. We used these thresholds to select for relevant and robust differentially expressed genes. Final annotation of the genome was defined based on the NCBI gene locus and the gene name and description of the reference *R. solanacearum* GMI1000 genome annotation (Supplementary Table 1).

**Table 1:** **Number and percentage of aligned reads to the *R. solanacearum* UY031 genome from mock-inoculated (Control) and inoculated *Solanum commersonii* accessions**.

			**BWA^a^**		**BWA_stringent^b^**		**Bowtie2_stringent**	
**Condition^c^**	**Replica**	**Total reads**	**Reads**	**%**	**Reads**	**%**	**Reads**	**%**
Resistant mock-inoculated	1	83867508	110859	0.1	66083	0.1	601	0.0
	2	88913944	42040	0.0	25296	0.0	771	0.0
Resistant infected	1	71855042	348369	0.5	330968	0.5	290036	0.4
	2	96470501	943974	1.0	924297	1.0	879112	0.9
	3	23473454	249285	1.1	234153	1.0	183728	0.8
Susceptible mock-inoculated	1	100234418	70173	0.1	40797	0.0	300	0.0
	2	27594608	15060	0.1	8889	0.0	137	0.0
Susceptible infected	1	75368620	249382	0.3	232550	0.3	211561	0.3
	2	93023963	2103356	2.3	2010284	2.2	1867585	2.0
	3	24695183	518872	2.1	484873	2.0	410525	1.7

a *Burrows-Wheeler Alignment*.

b *Burrows-Wheeler Alignment using stringent parameters as described in methods*.

c *Samples from Zuluaga, Solé, Lu, BMC Genomics, 2015*.

### Homology analysis

get_homologs (version 2.0; Contreras-Moreira and Vinuesa, 2013) was used for searching *R. solanacearum* UY031 homologous genes on *R. solanacearum* GMI1000, *R. solanacearum* IPO1609 and *R. solanacearum* UW551 strains as well as in *Pseudomonas syringae* pv. syringae B728a; NCBI RefSeq sequences GCF_001299555.1, GCF_000009125.1, GCF_001050995.1, GCF_000167955.1, and GCF_000012245.1, respectively. Default algorithm of bidirectional best-hits was used on homologous genes search.

### Functional categories

*R. solanacearum* UY031's genes were functionally categorized using two different strategies. Firstly, functional categories from *Pseudomonas syringae* pv. *syringae* B728a as defined by Yu et al. (2013), were translated to *R. solanacearum* UY031 based on homology information between the two strains. Although the *P. syringae*-derived categories should be more specific and accurate for another bacterial plant pathogen, almost 70% of the *R. solanacearum* UY031 genes could not be classified using this method. Therefore, a second strategy based on Clusters of Orthologous Groups (COG) categories was applied. Genome features were extracted from NCBI's RefSeq annotation and cdd2cog.pl script (version 0.1; Leimbach, 2016) was used to assign COG IDs and functional categories to the differentially expressed genes (Supplementary Table 1).

## Results

### Obtaining *R. solanacearum* sequences from infected root tissues

**Figure 1:**
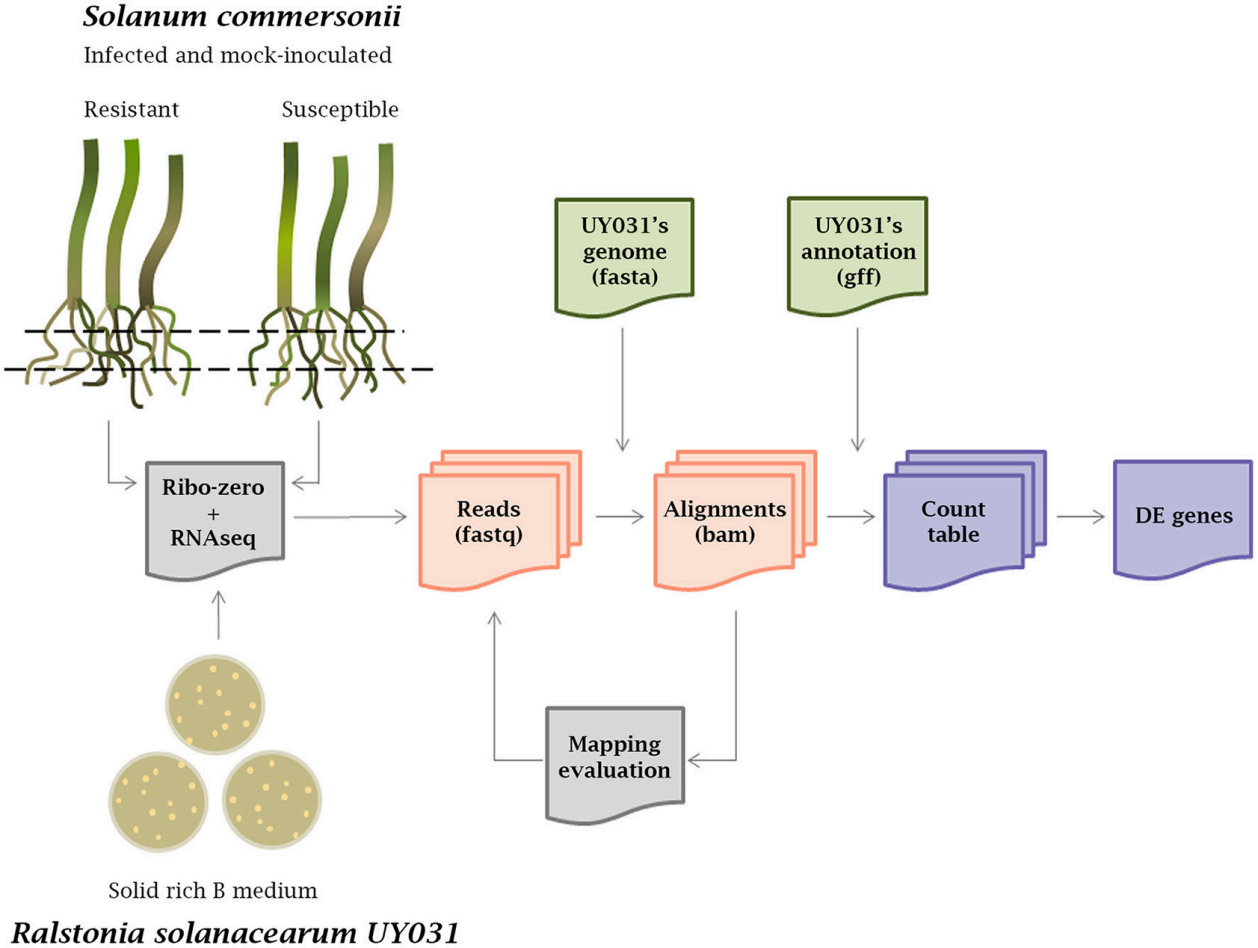
**Workflow of the transcriptomic analysis**. RNAseq was carried out from roots of infected and mock-inoculated *Solanum commersonii* resistant and susceptible varieties and from bacteria grown in solid rich B medium. Three biological replicates were used for each condition. Total extracted RNAs were treated with Ribo-zero to remove rRNA and sequenced using Illumina technology. Raw reads were aligned against the *R. solanacearum* UY031 genome using different alignment tools and mapping was visually evaluated with the IGV Browser. Mapped reads were quantified using count tables and differential expression (DE) analysis was carried out.

cDNA libraries from rRNA-depleted RNAs isolated from *S. commersonii* roots inoculated with *R. solanacearum* were sequenced using Illumina technology as previously reported (Zuluaga et al., 2015). To generate the transcriptomic profile of the bacteria growing inside root tissues, *R. solanacearum* UY031 sequences were obtained following the pipeline detailed in Figure 1. First, reads from mock-inoculated plants were used as a control to determine the best alignment tool to map against the *R. solanacearum* UY031 reference genome (Guarischi-Sousa et al., 2016; see material and methods). The Bowtie2 alignment tool with stringent parameters was used, as it retained a number of *R. solanacearum* reads in mock-inoculated plants that could be considered background levels compared to the high read numbers from inoculated samples (Table 1). All samples were analyzed with Bowtie2, including RNA-seq reads coming from *in vitro* grown bacteria. We determined that around 1% of the total sequenced reads from plant tissues corresponded to *R. solanacearum* and these were retained for further analyses. *S. commersonnii* sequences accounted on average for 63.15% of the total reads sequenced and the remaining reads corresponded mostly to contamination by other bacterial endophytes. The retrieved bacterial sequences were quantified and differentially expressed (DE) genes comparing the different conditions were determined. Total RNAs from infected *S. commersonii* enabled transcript quantification for over 96% of *R. solanacearum* UY031 predicted genes (4,609 out of the 4,778; Guarischi-Sousa et al., 2016).

### Similar *R. solanacearum* genes are differentially expressed upon infection of resistant and susceptible *S. commersonii* plants

In order to compare the *R. solanacearum* gene expression patterns during infection of resistant and susceptible wild potato plants, we analyzed separately the bacterial reads obtained from infected *S. commersonii* accessions F118 and F97, respectively. Surprisingly, only two out of the 4,609 genes for which expression was detected showed differential expression between the two genotypes. The differentially-expressed (DE) genes, RSUY_RS08455, and RSUY_RS16950, were both up-regulated in bacteria grown inside the resistant accession (Table 2). The first gene corresponds to an uncharacterized member of the MarR transcriptional regulator family, while the second encodes a hypothetical protein.

**Table 2:** ***R. solanacearum* UY031 genes differentially expressed in resistant vs. susceptible *S. commersonii***.

**UY031 NCBI locus^a^**	**UY031 Prokka locus^b^**	**GMI1000 locus^c^**	**Gene product**	**Log_2_FC**	**Adjusted *p*-value**
*RSUY_RS08455*	*RSUY_17320*	*RSc1295*	MarR family transcriptional regulator	2.37	0.0004
*RSUY_RS16950*	*RSUY_34650*	*RSp0403*	hypothetical protein	2.53	0.0017

a *According to R. solanacearum UY031 genome annotation available at GenBank (NCBI)*.

b *According to R. solanacearum UY031 genome annotation from Guarischi-Sousa et al. (2016)*.

c *According to the homology Supplementary Table 1*.

Since *R. solanacearum* showed extremely similar (>99.9%) transcriptional behavior during interaction with both *S. commersonii* accessions, bacterial reads from both accessions were treated as biological replicates in the rest of this study.

### *R. solanacearum* activates stress-related genes and shuts down metabolic activities during early root colonization

**Figure 2:**
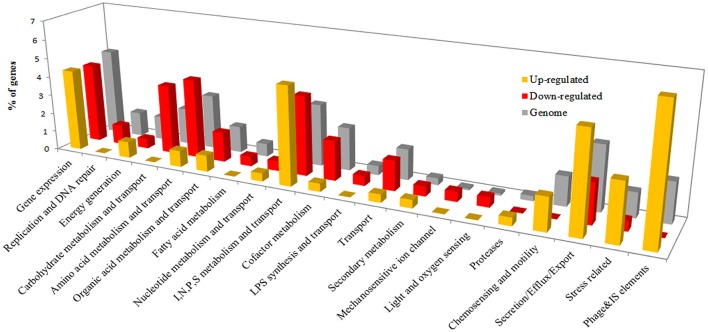
**Percentage of DE genes classified into *Pseudomonas syringae*-derived functional categories (Yu et al., 2013)**. Genes DE between growth *in planta* vs. rich medium were classified according to the functional categories described for *P. syringae* (Yu et al., 2013). Categories were grouped by function similarity for better visualization (Supplementary Table 4). As a reference, functional category distribution considering all annotated genes in the UY031 genome is shown.

The *R. solanacearum in planta* gene expression dataset was compared to a reference condition consisting of bacteria grown on solid rich B medium. Bacteria grown on solid medium were used as the reference condition instead of liquid cultures. *R. solanacearum* colonies grown on solid media better mimic the biofilms and microcolonies formed by *R. solanacearum* during early infection, when most bacteria occupy plant intercellular spaces (Mori et al., 2016). A total of 422 genes were differentially expressed during pre-symptomatic infection (231 up-regulated and 191 down-regulated), compared to growth on rich medium (Supplementary Table 2). These DE genes were classified into the functional categories previously used for gene expression studies in the plant pathogenic bacterium *P. syringae* (Yu et al., 2013; Supplementary Table 3). The number of successfully classified genes in each category was quantified in differentially induced or repressed groups and in the whole genome as a reference (Figure 2). This analysis revealed four categories highly over-represented in the up-regulated genes and under-represented in down-regulated genes: stress, secretion, chemosensing, and motility and phage and insertion sequences (IS). These categories represent together approximately 20% of the total induced genes *in planta*. The opposite trend (under-representation in up-regulated and over-representation in down-regulated genes) is observed in the categories including genes for transport and metabolism of amino acids and carbohydrates. In addition, the categories replication and DNA repair, transport, fatty acid metabolism and cofactor metabolism are strongly under-represented amongst the up-regulated genes *in planta* (Figure 2).

We used the *P. syringae* categories because they were created to describe the genes of a bacterial plant pathogen and are thus very informative for this study. However, the same analysis was carried out using the widely used but more general COG categories, and the results confirmed the previously-described tendencies (Supplementary Figure 1). Genes involved in carbohydrate, amino acid, lipid, cofactor, and secondary metabolism were over-represented among those down-regulated *in planta*. A clear enrichment of replication, cell motility and recombination and repair (where IS elements are included) was observed in the up-regulated genes. Interestingly, a clear asymmetry was seen for unclassified genes in this case, for they represent 40% of the up-regulated but only 7% of the down-regulated genes.

Closer scrutiny of the up-regulated genes in the plant revealed that the category secretion included 11 genes encoding the T3SS and its associated effectors and four chemosensing and motility genes, coding for pilus assembly and flagellum transcriptional activators (Table 3).

**Table 3:** ***R. solanacearum* UY031 genes differentially expressed in potato roots vs. solid rich medium**.

**Function**	**UY031 NCBI locus^a^**	**UY031 Prokka locus^b^**	**GMI1000 locus^c^**	**Log_2_FC**	**Gene name**	**Gene product**
***RALSTONIA SOLANACEARUM*** **VIRULENCE GENES**						
Type III secretion system and effectors	RSUY_RS19685	RSUY_40420	RSp0855	7.80	*hrpY*	Type III secretion system protein HrpY
	RSUY_RS19795	RSUY_40640	RSp0877	4.66	*popA*	Type III effector protein PopA
	RSUY_RS19790	RSUY_40630	RSp0876	4.27	*popB*	Type III effector protein PopB
	RSUY_RS20380	RSUY_41860	RSp1024	3.96	*awr5_1*	Type III effector protein AWR5
	RSUY_RS22080	RSUY_45370	RSp0900	3.94	*popF1*	Type III effector protein PopF1
	RSUY_RS16550	RSUY_33840	RSp0304	3.76	*ripD*	Type III effector protein RipD
	RSUY_RS19785	RSUY_40620	RSp0875	3.35	*popC*	Type III effector protein PopC
	RSUY_RS19735	RSUY_40520	RSp0865	3.08	*hrpK*	Type III secretion system protein HrpK
	RSUY_RS19690	RSUY_40430	RSp0856	2.86	*hrpX*	Type III secretion system protein HrpX
	RSUY_RS19770	RSUY_40590	RSp0872	2.69	*hrcT*	HrcT family type III secretion system export apparatus protein
	RSUY_RS09370	RSUY_19160	−	2.44	*ripV2*	Type III effector protein RipV2
	RSUY_RS19150	RSUY_39290	RSp0731	−2.61	*ripTPS*	Trehalose-6-phosphate synthase
	RSUY_RS21730	RSUY_44630	RSp1374	−2.88	*ripS2*	Type III effector protein SKWP2
	RSUY_RS21610	RSUY_44390	RSp1277	−3.50	*ripQ*	Type III effector protein RipQ
Motility	RSUY_RS04635	RSUY_09450	RSc0727	3.14	*pilV*	Type IV pilus modification protein PilV
	RSUY_RS22435	RSUY_46110	RSp1412	2.65	*flhC*	Transcriptional activator FlhC
	RSUY_RS02415	RSUY_04970	RSc2974	2.62	*pilN*	Tfp pilus assembly protein PilN
	RSUY_RS22440	RSUY_46120	RSp1413	2.47	*flhD*	Flagellar transcriptional activator FlhD
	RSUY_RS04630	RSUY_09440	RSc0726	2.30	*pilW*	Pilus assembly protein PilW
	RSUY_RS02410	RSUY_04960	RSc2975	2.28	*pilM*	Pilus assembly protein PilM
	RSUY_RS04330	RSUY_08850	RSc0668	2.20	*pilG*	Two-component system response regulator
	RSUY_RS04335	RSUY_08860	RSc0669	1.95	*pilH*	Two-component system response regulator
	RSUY_RS11250	RSUY_22930	RSc1986	1.24	*fimV*	Tfp pilus assembly protein FimV
	RSUY_RS04590	RSUY_09370	RSc0718	−3.56	*pilY*	Pilus assembly protein PilY
Stress responses	RSUY_RS22220	RSUY_45660	RSp1581	3.16	*katE*	Catalase katE
	RSUY_RS00425	RSUY_00900	RSc3398	3.10	*hmpX*	flavohemoprotein
	RSUY_RS17495	RSUY_35830	RSp0245	2.64	*ahpC1*	Peroxiredoxin
	RSUY_RS17500	RSUY_35840	RSp0246	2.21	*ahpF*	Alkyl hydroperoxide reductase subunit F
	RSUY_RS04770	RSUY_09720	RSc0754	1.96	−	Peroxidase
	RSUY_RS04870	RSUY_09930	RSc0775	1.82	*katGb*	Catalase katGb
	RSUY_RS01185	RSUY_02450	RSc3254	−3.11	−	alkyl hydroperoxide reductase
Other virulence factors	RSUY_RS18925	RSUY_38830	RSp0676	3.71	*metE*	Methionine synthase II (cobalamin-independent)
	RSUY_RS01465	RSUY_03030	RSp0693	3.38	*hdfA*	Dioxygenase
	RSUY_RS14015	RSUY_28660	RSc0408	2.22	*rpoN1*	RNA polymerase sigma-54 factor
	RSUY_RS17795	RSUY_36440	RSp1529	1.72	*efe*	2-oxoglutarate-dependentethylene/succinate-forming enzyme
	RSUY_RS04455	RSUY_09100	RSc0693	−2.19	*kdtA*	3-deoxy-D-manno-octulosonic-acid transferase
Type II Secretion System	RSUY_RS01675	RSUY_03470	RSc3109	−2.63	*gspJ*	General secretion pathway protein GspJ
	RSUY_RS16275	RSUY_33280	RSp0148	−3.19	*gspE*	General secretion pathway protein GspE
Type VI secretion system	RSUY_RS19215	RSUY_39440	RSp0746	2.25	−	Type VI secretion protein
Cofactor metabolism and transport	RSUY_RS04050	RSUY_08280	RSc2633	−2.92	*pabB*	aminodeoxychorismate synthase component I
Quorum sensing	RSUY_RS01010	RSUY_02100	RSc3286	−3.24	*solI*	Acyl-homoserine-lactone synthase
***RALSTONIA SOLANACEARUM*** **GENES INVOLVED IN PLANT COLONIZATION**						
Aminoacid metabolism	RSUY_RS21930	RSUY_45070	RSp1263	2.019462	*nadB2*	L-aspartate oxidase
	RSUY_RS04790	RSUY_09760	RSc0758	1.900185	−	Tryptophan 2,3-dioxygenase 1
	RSUY_RS01705	RSUY_03530	RSc3103	−1.93219	−	4-hydroxyphenylpyruvate dioxygenase
	RSUY_RS00955	RSUY_01990	RSc3295	−1.97096	*gcvP*	glycine dehydrogenase
	RSUY_RS00965	RSUY_02010	RSc3293	−2.1387	*gcvT*	aminomethyltransferase
	RSUY_RS08880	RSUY_18160	RSc1381	−2.22786	−	glutathione ABC transporter permease GsiC
	RSUY_RS02965	RSUY_06080	RSc2867	−2.45751	*dppD1*	peptide ABC transporter substrate-bindingprotein
	RSUY_RS19000	RSUY_38980	RSp0691	−2.63642	*hmgA*	homogentisate 1,2-dioxygenase
	RSUY_RS08860	RSUY_18120	RSc1377	−2.72373	−	transcriptional regulator
	RSUY_RS00950	RSUY_01980	RSc3296	−2.82715	*sdaA2*	L-serine ammonia-lyase / L-serine ammonia-lyase
	RSUY_RS18995	RSUY_38970	RSp0690	−2.93699	*hmgB*	fumarylacetoacetase
	RSUY_RS08895	RSUY_18190	RSc1384	−3.10843	−	D-aminopeptidase
	RSUY_RS08865	RSUY_18130	RSc1378	−3.44342	−	isoaspartyl peptidase
Carbohydrate metabolism	RSUY_RS22935	RSUY_47230	RSp1633	−1.90617	*xylF*	D-xylose ABC transporter substrate-bindingprotein
	RSUY_RS22945	RSUY_47250	RSp1635	−2.40016	*xylH*	xylose ABC transporter permease
	RSUY_RS21965	RSUY_45140	RSp1270	−2.4781	−	glycosyl hydrolase
	RSUY_RS22940	RSUY_47240	RSp1634	−2.98048	*xylG*	D-xylose ABC transporter ATP-binding protein
	RSUY_RS17060	RSUY_34910	RSp0423	−3.65067	−	aldolase
	RSUY_RS22950	RSUY_47260	RSp1636	−4.75552	−	NAD-dependent dehydratase
Transcriptional and response regulators	RSUY_RS08455	RSUY_17320	RSc1295	4.365537	−	MarR family transcriptional regulator
	RSUY_RS22955	RSUY_47270	RSp1637	−1.62836	−	LacI family transcriptional regulator
	RSUY_RS06090	RSUY_12470	RSc2209	−1.75486	−	LysR family transcriptional regulator
	RSUY_RS00225	RSUY_00480	RSc0040	−2.26596	−	two-component system response regulator
	RSUY_RS00910	RSUY_01900	RSc3301	−2.57757	*putA*	trifunctional transcriptional regulator
Siderophore biosynthesis	RSUY_RS17055	RSUY_34900	RSp0422	−2.30463	−	siderophore biosynthesis protein
	RSUY_RS03590	RSUY_07350	RSc2729	−2.4997	−	membrane protein / membrane protein
	RSUY_RS17040	RSUY_34870	RSp0419	−2.75083	−	siderophore biosynthesis protein
	RSUY_RS17050	RSUY_34890	RSp0421	−2.94313	−	siderophore biosynthesis protein
Nitrogen metabolism	RSUY_RS14025	RSUY_28680	RSc0406	2.27	*ptsN*	PTS IIA-like nitrogen-regulatory protein PtsN
	RSUY_RS17995	RSUY_36860	RSp0980	2.24	*narL*	DNA-binding response regulator
	RSUY_RS11470	RSUY_23380	RSc2031	−3.3167	*ureE*	urease accessory protein UreE
Transporters	RSUY_RS20760	RSUY_42660	RSp1283	2.005142	−	porin
	RSUY_RS11090	RSUY_22610	RSc1951	−2.22	−	cation acetate symporter
	RSUY_RS22930	RSUY_47220	RSp1632	−2.42488	*oprB*	porin
	RSUY_RS05795	RSUY_11880	RSc2274	−2.96894	*ragC*	Cation efflux protein
Organic acid metabolism	RSUY_RS19540	RSUY_40110	RSp0826	3.403389	−	5-dehydro-4-deoxyglucarate dehydratase
	RSUY_RS19480	RSUY_39990	RSp0814	2.44631	*mqo*	malate:quinone oxidoreductase
	RSUY_RS11960	RSUY_24410	RSc2358	−1.7429	*ppc*	phosphoenolpyruvate carboxylase
Proteases	RSUY_RS12475	RSUY_25460	RSc2465	2.359347	*clpS*	ATP-dependent Clp protease adaptor ClpS
	RSUY_RS18550	RSUY_38040	RSp0603	2.211049	−	serine protease
	RSUY_RS14120	RSUY_28870	RSc0388	−1.98903	−	zinc protease
Lipid metabolism	RSUY_RS17295	RSUY_35410	−	2.478807	−	Acyl-CoA synthetase
	RSUY_RS01975	RSUY_04090	RSc3052	−2.40887	*glpK*	glycerol kinase
Energy	RSUY_RS08510	RSUY_17430	RSc1305	3.640811	*fpr*	ferredoxin–NADP(+) reductase
	RSUY_RS08360	RSUY_17120	RSc1276	3.417949	−	cytochrome c oxidase, cbb3-type subunit I
Signal transduction	RSUY_RS23055	RSUY_47460	RSc0617	1.988909	−	signal peptidase
	RSUY_RS01700	RSUY_03520	RSc3104	1.73953	−	calcium sensor EFh
Stress related	RSUY_RS13090	RSUY_26760	RSc0582	2.610905	−	avrD-like protein
	RSUY_RS21705	RSUY_44580	RSp1306	−2.11404	*speE2*	spermidine synthetase
Cofactor metabolism and transport	RSUY_RS02845	RSUY_05840	RSc2886	−2.57058	−	adenylate cyclase
Recombination and repair	RSUY_RS07890	RSUY_16140	RSc1189	−2.09233	−	recombinase RecB
Translation	RSUY_RS15445	RSUY_31580	RSc0085	−2.70405	*cca*	multifunctional CCA protein
Hypothetical proteins	RSUY_RS19605	RSUY_40240	−	4.758974	−	hypothetical protein
	RSUY_RS17575	RSUY_35990	RSp0261	4.146986	−	membrane protein
	RSUY_RS16950	RSUY_34650	RSp0403	3.74634	−	hypothetical protein
	RSUY_RS22040	RSUY_45290	RSp0982	3.589851	−	hypothetical protein
	RSUY_RS12885	RSUY_26330	RSc0613	3.409348	−	hypothetical protein
	RSUY_RS08290	RSUY_16960	RSc1262	3.394474	−	hypothetical protein
	RSUY_RS06775	RSUY_13900	RSc0971	3.025981	−	hypothetical protein
	RSUY_RS20375	RSUY_41850	−	3.019263	−	hypothetical protein
	RSUY_RS01105	RSUY_02290	RSc3270	2.806237	−	hypothetical protein
	RSUY_RS15470	RSUY_31630	RSc0080	2.792044	−	hypothetical protein
	RSUY_RS14600	RSUY_29830	RSc0297	2.641501	−	hypothetical protein
	RSUY_RS05760	RSUY_11810	RSc2280	2.552951	−	hypothetical protein
	RSUY_RS10215	RSUY_20850	RSc1622	2.233892	−	hypothetical protein
	RSUY_RS12820	RSUY_26200	−	2.213444	−	hypothetical protein
	RSUY_RS22015	RSUY_45240	RSp1546	2.185718	−	hypothetical protein
	RSUY_RS01435	RSUY_02950	RSc0616	2.148496	−	hypothetical protein
	RSUY_RS06705	RSUY_13730	RSc0953	2.116755	−	hypothetical protein
	RSUY_RS01875	RSUY_03890	RSc3072	2.098485	−	hypothetical protein
	RSUY_RS04190	RSUY_08560	RSc2555	1.909667	−	membrane protein
	RSUY_RS05940	RSUY_12170	RSc2238	1.775776	−	hypothetical protein
	RSUY_RS04990	RSUY_10170	RSc0799	−1.56204	−	hypothetical protein
	RSUY_RS02135	RSUY_04410	RSc3030	−2.05111	−	hypothetical protein
	RSUY_RS20585	RSUY_42270	−	−2.22451	−	membrane protein
	RSUY_RS14980	RSUY_30600	RSc0211	−2.4077	−	membrane protein
	RSUY_RS17045	RSUY_34880	RSp0420	−2.58002	−	membrane protein
	RSUY_RS15175	RSUY_31000	RSc0146	−2.83465	−	hypothetical protein
**PUTATIVE VIRULENCE GENES AND PLANT COLONIZATION METABOLIC ACTIVITIES**						
Transporters	RSUY_RS00490	RSUY_01050	RSc3386	3.50	−	metal ABC transporter substrate-binding protein
	RSUY_RS17605	RSUY_36050	RSp0429	3.24	−	MFS transporter
	RSUY_RS20020	RSUY_41100	RSp0931	2.92	−	ABC transporter
	RSUY_RS19045	RSUY_39070	RSp0706	−1.77	−	metal-dependent hydrolase
	RSUY_RS18205	RSUY_37320	RSp0481	−2.03	−	ABC transporter substrate-binding protein
	RSUY_RS18195	RSUY_37300	RSp0479	−2.09	−	amino acid ABC transporter ATPase
	RSUY_RS21220	RSUY_43600	RSp1181	−2.11	−	transporter
	RSUY_RS18895	RSUY_38770	RSp0670	−2.14	−	acriflavine resistance protein B / transporter protein
	RSUY_RS17425	RSUY_35690	RSp0234	−2.37	−	MFS transporter
	RSUY_RS09885	RSUY_20190	RSc1738	−2.40	−	ABC transporter ATPbinding protein
	RSUY_RS21615	RSUY_44400	RSp1278	−2.49	−	MFS transporter
	RSUY_RS21315	RSUY_43800	RSp1200	−2.60	−	RND transporter
	RSUY_RS03020	RSUY_06190	RSc2856	−2.72	−	MFS transporter
	RSUY_RS01560	RSUY_03230	RSc3134	−2.94	−	MFS transporter
	RSUY_RS06940	RSUY_14230	RSc1002	−2.95	−	membrane protein
	RSUY_RS15855	RSUY_32410	−	−2.98	*oprM*	RND transporter
	RSUY_RS20395	RSUY_41890	−	−2.99	*ybtP*	ABC transporter ATP-binding protein
	RSUY_RS15985	RSUY_32660	RSp0078	−3.05	−	MFS transporter
	RSUY_RS18200	RSUY_37310	RSp0480	−3.17	−	amino acid ABC transporter permease
	RSUY_RS19050	RSUY_39080	RSp0707	−3.19	−	ABC transporter ATP-binding protein
	RSUY_RS21055	RSUY_43260	RSp1114	−3.35	−	RND transporter
	RSUY_RS01890	RSUY_03920	RSc3069	−3.50	−	MFS transporter
	RSUY_RS22255	RSUY_45730	RSp1595	−3.73	−	ABC transporter ATP-binding protein
	RSUY_RS04055	RSUY_08290	RSc2632	−3.85	−	ABC transporter ATP-binding protein
Lipid metabolism	RSUY_RS14075	RSUY_28780	RSc0396	3.29	*ipk*	4-diphosphocytidyl-2C-methyl-D-erythritolkinase
	RSUY_RS10705	RSUY_21830	RSc1540	3.14	−	acyltransferase
	RSUY_RS19325	RSUY_39670	−	2.15	−	Phosphatidylserine/phosphatidylglycerophosphate/cardiolipin synthase
	RSUY_RS09620	RSUY_19650	RSc1772	−2.06	−	alpha/beta hydrolase
	RSUY_RS14790	RSUY_30210	RSc0262	−2.17	−	glyoxylate/hydroxypyruvate reductase A
	RSUY_RS00675	RSUY_01440	RSc3346	−2.24	−	alpha/beta hydrolase
	RSUY_RS13905	RSUY_28430	RSc0427	−2.30	−	beta-ketoacyl-[acyl-carrier-protein] synthaseII
	RSUY_RS09090	RSUY_18590	−	−2.77	−	Lysophospholipase
	RSUY_RS01035	RSUY_02150	RSc3283	−2.82	*glxR*	2-hydroxy-3-oxopropionate reductase
	RSUY_RS10955	RSUY_22330	RSc1874	−2.82	−	NUDIX hydrolase
	RSUY_RS14265	RSUY_29170	RSc0357	−2.86	*gpsA*	glycerol-3-phosphate dehydrogenase (NAD(P)(+))
	RSUY_RS11775	RSUY_23990	RSc2091	−3.04	−	ABC transporter permease
	RSUY_RS15945	RSUY_32580	RSp0036	−3.05	−	acyl-CoA dehydrogenase
	RSUY_RS21855	RSUY_44890	RSp1245	−3.30	−	esterase
	RSUY_RS20415	RSUY_41930	−	−3.31	−	Acyl-coenzyme A synthetase
	RSUY_RS21590	RSUY_44350	−	−3.32	−	Dehydrogenases
	RSUY_RS20425	RSUY_41950	−	−3.60	−	Polyketide synthase
	RSUY_RS14720	RSUY_30070	RSc0275	−3.61	−	short-chain dehydrogenase
	RSUY_RS10105	RSUY_20630	RSc1643	−4.27	*ispD*	2-C-methyl-D-erythritol 4-phosphatecytidylyltransferase
Other putative virulence factors	RSUY_RS16085	RSUY_32860	RSp0112	2.09	−	carbonic anhydrase
	RSUY_RS00735	RSUY_01550	RSp0085	1.66	−	type IV secretion protein Rhs
	RSUY_RS00905	RSUY_01890	RSc3302	−2.04	*priA*	primosomal protein N'
	RSUY_RS15535	RSUY_31760	RSc0068	−2.06	*smf*	DNA processing protein DprA
	RSUY_RS14955	RSUY_30550	RSc0222	−2.29	*rtcR*	Fis family transcriptional regulator
	RSUY_RS17190	RSUY_35180	RSp0181	−3.10	−	activator of HSP90 ATPase
	RSUY_RS14940	RSUY_30520	RSc0226	−3.23	*rtcA*	RNA 3'-terminal phosphate cyclase
Sulfur metabolism and transport	RSUY_RS12265	RSUY_25040	RSc2425	3.22	*cysI1*	Sulfite reductase/sulfite reductase
	RSUY_RS07020	RSUY_14390	RSc1019	2.23	*nifS*	Cysteine desulfurase IscS
	RSUY_RS12250	RSUY_25010	RSc2422	2.14	*cysD*	Sulfate adenylyltransferase small subunit
	RSUY_RS12245	RSUY_25000	RSc2421	1.89	*cysN*	Sulfate adenylyltransferase
	RSUY_RS07025	RSUY_14400	RSc1020	1.60	*nifU*	Iron-sulfur cluster scaffold-like protein
	RSUY_RS17845	RSUY_36540	RSp1519	−3.36	−	Membrane protein
Cofactor metabolism and transport	RSUY_RS11705	RSUY_23850	RSc2077	1.85	*ilvI*	acetolactate synthase
	RSUY_RS18660	RSUY_38270	RSp0615	−2.97	*cbiA*	cobyrinic acid a,c-diamide synthase
	RSUY_RS18690	RSUY_38330	RSp0621	−2.97	*cbiL*	precorrin-2 C(20)-methyltransferase
	RSUY_RS18680	RSUY_38310	RSp0619	−3.00	*cbiG*	cobalamin biosynthesis protein CbiG
	RSUY_RS03905	RSUY_07990	RSc2663	−3.51	−	ATP:cob(I)alamin adenosyltransferase
Phosphate mobilization	RSUY_RS10765	RSUY_21950	RSc1529	2.01	*pstS1*	phosphate ABC transporter substrate-bindingprotein PstS
	RSUY_RS07715	RSUY_15790	RSc1160	1.63	*suhB*	Inositol monophosphatase
	RSUY_RS10750	RSUY_21920	RSc1532	1.52	*pstB*	phosphate ABC transporter ATP-binding protein

a *According to R. solanacearum UY031 genome annotation available at GenBank (NCBI)*.

b *According to R. solanacearum UY031 genome annotation from Guarischi-Sousa et al. (2016)*.

c *According to the homology Supplementary Table 1*.

Taken together, these results show a major induction of stress-related activities and an inhibition of the central metabolism when the bacterium grows *in planta* compared to synthetic media.

### *R. solanacearum* virulence genes are differentially expressed in wild potato roots

Among the 422 genes DE during *S. commersonii* root colonization, 34% (80 induced and 65 repressed genes) had been identified in previous studies analyzing gene expression of *R. solanacearum* cells recovered from infected plant stems (see references below). Notably, 73 genes were also DE in microarray analyses of *R. solanacearum* UW551 -a phylotype IIB strain highly similar to UY031- isolated from tomato (Jacobs et al., 2012). Also, 42 genes have been shown to be induced in a temperature-dependent manner when bacteria grew in tomato xylem or rhizosphere (Bocsanczy et al., 2014; Meng et al., 2015). In addition, 31 DE genes (most of them induced *in planta*) are part of either the HrpB or HrpG regulons, which are known to trigger expression of the T3SS and other virulence genes in response to direct plant cell contact (Valls et al., 2006).

Amongst the *R. solanacearum* genes induced during plant colonization, 31 encode already reported virulence traits (Table 3). As expected, genes encoding the T3SS (*hrpY, hrpX, hrpK, hrcT)* and some of its related effectors *(ripV2, popC*, ripD, *popF1, awr5_1, popB*, and *popA*) were induced inside the plant (Boucher et al., 1987; Cunnac et al., 2004). Motility and adherence genes were also up-regulated, including type IV pili (*pilG, pilH, pilN, pilM, pilY, pilW*, and *fimV*), as well as the transcriptional activators of the flagellum genes *flhC* and *flhD* (Kang et al., 2002; Tans-Kersten et al., 2004). Other induced genes encoding described factors that are key for bacterial virulence included *hdfA* (Delaspre et al., 2007), *efe* (Valls et al., 2006), *metE* (Plener et al., 2012), and *rpoN1* (Lundgren et al., 2015; Ray et al., 2015; Table 3). Peroxidases, catalases (*katE, katG*) and alkyl hydroperoxide reductases (*ahpC1, ahpF*), which have been described to combat the oxidative stress response during plant infection (Rocha and Smith, 1999; Flores-Cruz and Allen, 2009; Ailloud et al., 2016) were also induced. Similarly, the flavohemoprotein *hmpX*, involved in NO-detoxification (Dalsing and Allen, 2014), was also induced.

In contrast, only 10 reported virulence determinants were down-regulated, including the type III effectors *ripQ, ripS2*, and *ripTPS*, the quorum sensing regulator *solI* (Flavier et al., 1997) and the Type II secretion system genes *gspE, gspJ* (Table 3).

### *R. solanacearum* genes for plant colonization are differentially expressed in *S. commersonii* roots

Thirty-six *R. solanacearum* genes previously described as related to plant colonization in gene expression studies in other plant species were also induced in potato. Few metabolic genes were induced *in planta*, being an exception *nadB2*, involved in the degradation of L-aspartate in the xylem (Brown and Allen, 2004) and the *ptsN* and *narL* nitrogen metabolism genes, known to be active during plant colonization (Dalsing and Allen, 2014; Dalsing et al., 2015; Table 3).

Amongst the down-regulated genes, 42 had also been described as specifically down-regulated during plant colonization (Jacobs et al., 2012). Most repressed genes encoded metabolic enzymes and transporters. Examples are the xylose transporters *xylF, xylG*, and *xylH*, glycine catabolism genes *gcvP, gcvT*, and *gcvA*, the adenilate cyclase coding gene RSUY_RS02845, four siderophore biosynthesis genes and 11 genes involved in amino acid metabolism (Table 3). Also, the stress response gene *speE2* and five transcriptional and response regulators were repressed *in planta*.

### Novel putative virulence genes and metabolic traits involved in early stages of wild potato infection by *R. solanacearum*

Transcriptomic analysis of *S. commersoni* early root infection revealed highly induced *R. solanacearum* virulence factors still uncharacterized in this pathogen that may play a role at this stage of the interaction with the host. An example of this is *suhB*, a global virulence regulator controlling the type III and type VI secretion systems, flagellum biosynthesis, and biofilm formation in the human pathogens *Burkholderia cenocepacia* and *Pseudomonas aeruginosa* (Rosales-Reyes et al., 2012; Li et al., 2013). Similarly, a *P. aeruginosa* orthologue of the *in planta* induced type IV secretion gene Rhs has been described as a virulence determinant (Kung et al., 2012).

Metabolic traits that might be key at this point of plant infection are the assimilatory sulfate reduction pathway and phosphate mobilization, since c*ysD, cysN*, and *cysI* (sulfate reduction) and *pstB* and *pstS1* (phosphate mobilization) were induced during *S. commersonii* root infection. Also, carbonic anhydrase (*RSUY_RS16085*), which plays a role in disease establishment between potato and *Phytophthora infestans* (Restrepo et al., 2005), was also found to be up-regulated in the *R. solanacearum* interaction with wild potato.

The most important category amongst the *R. solanacearum* genes down-regulated in *S. commersonii* with so far no assigned functions in plant colonization or virulence was metabolite transporters. Almost half of these corresponded to the ABC-family, including five amino acid transporters. In contrast, the seven major facilitator superfamily (MFS) transporters found in this category are involved with carbohydrate transport. The rest of genes were classified as permeases or RND (Resistance-Nodulation-Division) efflux systems (Table 3). The major metabolic activities identified as repressed in planta for the first time were lipid mobilization and cofactor metabolism, such as the anaerobic cobalamin biosynthesis operon (*cbiA, cbiG*, and *cbiL*), and stress-response genes such as *rtcA* and *rtcR*, involved in RNA repair (Das and Shuman, 2013).

In sum, our work reflects important gene expression changes between parasitic life and growth in rich medium (see below). This was corroborated by the fact that seven genes annotated as response regulators were also DE, five of them induced (Table 3).

## Discussion

### Some *R. solanacearum* virulence and stress-responsive genes are induced irrespective of the plant host

1/3 of the *R. solanacearum* genes DE during potato infection had been also found DE when the bacterium colonized other plant species and many of these correspond to virulence determinants. For instance, we found that genes encoding the type III secretion system and its associated effectors (*popA, popB, popC, popF1, ripD, ripV2*, and *awr5_1*) were induced in potato (Table 3). Except for *awr5_1*, all these effectors had already been described as up-regulated when the bacterium grew in tomato and in melon (Ailloud et al., 2016), likely indicating that they are part of the minimal gene set required for bacterial virulence. Similarly, the effector *ripTPS* was down-regulated both in potato (Table 3) and during the interaction with melon (Ailloud et al., 2016). Also sharing similar up-regulation in potato (Table 3) and tomato are the transcriptional activators *flhC* and *flhD* (Jacobs et al., 2012), which regulate flagellum-encoding genes (Tans-Kersten et al., 2004) and the nitrogen metabolism genes *narL, ptsN*, and *hmpX* (Dalsing and Allen, 2014; Dalsing et al., 2015), implying that they all play a key role during plant infection. Additional genes induced during potato colonization had been described as key for virulence on other plant hosts, including small molecule *hdfA* (Delaspre et al., 2007), the ethylene forming enzyme *efe* (Valls et al., 2006), the methionine metabolism gene *metE* (Plener et al., 2012) and the alternative sigma factor *rpoN1* (Lundgren et al., 2015; Ray et al., 2015). These factors may be also considered essential for growth *in planta*, irrespective of the infected species.

Several transposable elements had been identified in an *in vivo* screening for genes expressed during *R. solanacearum* growth in tomato plants (Brown and Allen, 2004), and we found 16 transposases up-regulated in potato (Table 3). This may reflect common stressing conditions in various plant hosts, as stress is known to turn on transcription of transposable elements in various organisms (Capy et al., 2000). Oxidative stress seems also a condition generally encountered by *R. solanacearum* in plant tissues, as peroxidases, catalases, and peroxiredoxins, required for the bacterium to combat this stress in different plants (Rocha and Smith, 1999; Flores-Cruz and Allen, 2009; Ailloud et al., 2016), were also induced in potato.

### Changes in the host environment and/or the disease stage may account for opposing bacterial virulence gene expression in different plants

Some of the *R. solanacearum* virulence genes DE in potato showed opposite trends in other host plants. *ripQ* and *ripS2, two* of the three type III secreted effectors inhibited in potato were, respectively, upregulated and not DE in melon, tomato and banana (Ailloud et al., 2016). Interestingly, these two downregulated effectors, together with the also repressed stress response gene *speE2*, are located in a genomic region that is deleted in the avirulent *R. solanacearum* strain UY043 (Siri et al., 2014), which suggests their involvement in bacterial virulence. Similarly, the effector *awr5_1*, which was described to trigger hypersensitive response (HR) in tobacco and to inhibit the TOR pathway (Sole et al., 2012; Popa et al., 2016b), showed opposite regulation in potato when compared to tomato and melon (Ailloud et al., 2016), suggesting that it may play host-specific roles. Similarly, genes *pilG, pilH, pilN, pilM, pilY*, and *pilW*, coding for structural components of the type IV pili involved in twitching motility and adherence (Liu et al., 2001; Kang et al., 2002) were induced in the current work but repressed in other plant species (Jacobs et al., 2012).

In addition, some virulence determinants well-described as induced during growth *in planta* were repressed or not DE in potato. Remarkably, the exopolysaccharide synthesis and regulation genes (*eps*) as well as most known cell wall degrading enzymes (*pehA, pehB, pehC, egl*, and *cbhA*), which are virulence determinants (Schell, 2000) induced during tomato infection (UW551 strain) infection (Jacobs et al., 2012) were absent from the potato DE dataset.

Differences in the host environment or in the tissue environment and disease stage are the two most plausible reasons for the discrepancies between virulence gene expression data in potato and in other plant hosts. We favor the latter explanation, as our samples were collected from bacteria growing in the root (including apoplastic and xylematic bacteria) at early times after inoculation while all previous transcriptomic work had been performed from bacteria extracted from xylem at later infection stages.

Three independent observations support the existence of stage-specific environmental cues that differentially affect gene expression in this work compared to previous studies. First, genes that are induced at high bacterial densities are absent from the potato DE genes. Examples are the mentioned exopolysaccharide synthesis genes or the quorum sensing regulator *solI*, repressed in our conditions but slightly induced in bacteria isolated from the tomato shoot xylem (Jacobs et al., 2012). In the low bacterial cell densities in the roots the *phcA* cell-density regulator was not induced, impeding *solI* or *eps* expression (Huang et al., 1995; Flavier et al., 1997). Second, three out of the six type III effectors that are induced in potato were described as secreted at early stages (Lonjon et al., 2016), two of them (*popF1* and *popA*) also proposed to play an important role in the first steps of infection (Kanda et al., 2003). On the contrary, only two out of the 38 described as “late” effectors (*ripD* and *popC*) were induced in our root transcriptome. Third, the afore-mentioned transcriptional regulators *flhC* and *flhD* responsible for the activation of the flagellum genes were up-regulated in potato root samples (Table 3) and also in the tomato xylem (Jacobs et al., 2012), but only in the latter were the flagellum structural genes induced, suggesting that the potato transcriptome represents an earlier stage where complete activation of this regulon has not yet occurred. These observations imply that our transcriptome represents a snapshot of a precise stage of the genetic programs deployed consecutively during plant colonization.

Finally, we cannot rule out that changes in *R. solanacearum* DE genes in different studies are due to the use of different strains. Differing transcriptomes of two *R. solanacearum* strains in the same plant environment have already been reported (Ailloud et al., 2016). However, the fact that previous gene expression studies were performed with strain UW551, which is genetically extremely close to UY031 used here, render this explanation unlikely. Standardization of the plant inoculation and sampling procedures and a systematic analysis of plant-pathogen interactions dissecting gene expression over time in a defined strain-host pathosystem would clarify the nature of the observed discrepancies between transcriptomic studies.

### The *R. solanacearum* metabolic state during potato root colonization

From the transcriptomic information gathered in this work, we can infer for the first time the environmental conditions encountered by *R. solanacearum* in the root, the site where plant infection takes place.

A first observation is that the bacterium seems to start to run out of O_2_. An indication of this is the highly induced *Cbb3*-cco, a high affinity cytochrome c oxidase known to contribute to the growth of *R. solanacearum* and other bacteria in microaerobic or anoxic environments (Colburn-Clifford and Allen, 2010; Hamada et al., 2014), such as the plant xylem (Pegg, 1985). Upregulation of the low O_2_ affinity cytochrome ubiquinol oxidase genes *cyoA1* and *cyoB1* reinforces the notion of a microaerobic rather than an anoxic environment. In agreement with this, *nrdB*, which is required for growth in aerobiosis (Casado et al., 1991), was up-regulated, and *nrdG* and *nrdD*, required in strict anaerobiosis (Garriga et al., 1996; Ailloud et al., 2016) were not induced. Further, the *cbiA, cbiL*, and *cbiG* genes, which are involved in anaerobic cobalamin synthesis (Roessner and Scott, 2006), were repressed. Another indication of microaerobic conditions is the induction of genes driving nitrate and sulfate anaerobic respiration. Examples are the *cys* genes, involved in the assimilatory sulfate reduction pathway (Kredich, 1992), *ptsN*—a nitrogen-dependent regulatory protein, *rpoN1*, -the global nitrogen regulator- and *narL* -the nitrate/nitrite-responsive transcriptional regulator- were all induced in wild potato roots. All these findings suggest that during early root infection *R. solanacearum* is experiencing the transition from an aerobic environment to the anaerobic conditions established at the onset of disease during xylem colonization (Ailloud et al., 2016).

Another take home message from the root transcriptomes is that few central metabolic pathways seem to be active. It was previously described that a large proportion of the *R. solanacearum* genes involved in amino acid metabolism and transport was down-regulated during growth in the xylem (Ailloud et al., 2016) and we found that this was also the case during growth in the root tissues at early stages of infection. For instance, the glycine catabolism genes *gcvP, gcvT*, and *gcvA* as well as the dipeptide uptake gene *dppD1* were repressed in both cases (Table 3; Ailloud et al., 2016). Other *R. solanacearum* metabolic genes previously known to be repressed *in planta* also down-regulated here included carbohydrate metabolism genes such as the xylose transporter operon *xylFGH* and Glucosamine 6-phosphate synthetase, the key enzyme controlling amino sugar biosynthesis (Milewski, 2002; Jacobs et al., 2012). Lipid metabolism was also strikingly repressed during root colonization. Out of the 21 DE genes involved in lipid mobilization, only 2 have been found in previous gene expression studies in *R. solanacearum* (Table 3; Jacobs et al., 2012). Thus, the downregulation of lipid metabolism could be specific to early infection stages or to wild potato colonization. In this sense, lipid metabolism has been reported to play an important role during plant-host interactions by modulating defense responses in plants and pathogen infection (Casadevall and Pirofski, 2001; Wenk, 2006). Cofactor metabolism was also repressed including the folate synthesis gene *pabB* (Table 3), already known to be down-regulated *in planta* (Shinohara et al., 2005), the cobalamin biosynthesis genes and adenilate cyclase. Repression of adenylate cyclase, which is a global metabolic regulator in bacteria (Ullmann and Danchin, 1980), illustrates the magnitude of the metabolic shutdown experienced by *R. solanacearum* in the roots of *S. commersonii*.

In contrast with the global metabolic shutdown, aspartate and tryptophan catabolism genes were up-regulated when *R. solanacearum* grew in the plant roots. The aspartate catabolism gene *nadB2* had already been identified as an essential gene for in planta growth in an *in vivo* screening (Brown and Allen, 2004). Interestingly, aspartate is the second most abundant aminoacid in the tomato apoplast and less so in the xylem (Zuluaga et al., 2013), which is in agreement with the bacterium mostly thriving in the apoplastic root spaces at the early infection times analyzed. Also induced was the Tryptophan 2,3-dioxygenase. Concentrations of this aminoacid are high at lateral root emergence sites (Jaeger et al., 1999), and it was suggested that it is also present in the tomato apoplast (Yu et al., 2013). Induction of tryptophan catabolism would thus be indicative of early plant colonization.

These results likely indicate the existence of a trade-off between the expression of virulence and metabolic genes. This has already been described in a previous study where the quorum-sensing-dependent regulatory protein PhcA regulated a trade-off between production of *R. solanacearum* exopolysaccharides and bacterial proliferation (Peyraud et al., 2016).

### Proposed new virulence determinants important for early root colonization

*RSUY_RS08455* and *RSUY_RS16950* were found to be upregulated in a resistant *S. commersonii* accession compared to a susceptible one (Table 2), as well as during root colonization compared to rich medium (Table 3). Although these genes also appeared in the microarray transcriptome of bacteria extracted from infected tomato xylem vessels (Jacobs et al., 2012), they have not been characterized.

Similarly, the gene encoding an avrD-like protein was up-regulated in tomato xylem (Jacobs et al., 2012) and in wild potato (Table 3). AvrD is required in *P. syringae* for the synthesis of syringolide, small molecules that can elicit a hypersensitive response on resistant plants (Keen et al., 1990; Mucyn et al., 2014). In *R. solanacearum* the avrD-like protein encoding gene is activated by the master virulence regulator HrpG (Valls et al., 2006). Considering the persistence of these three genes among the up-regulated during plant colonization, we suggest that they encode for potential virulence factors, probably necessary independently of the host or the infection stage.

Three genes found up-regulated in *S. commersonii* (*suhB, rhs* and the carbonic anhydrase gene *RSUY_RS16085*, Table 3) have been involved in bacterial virulence on animals and constitute putative virulence genes in *R. solanacearum*. Although classified as a phosphate mobilization gene (Table 3), *suhB* is a super-regulator involved in the proper rRNA folding (Singh et al., 2016). It plays a role in virulence of animal bacterial pathogens, influencing T3SS, T6SS, flagellum and biofilm regulation and probably acts in opposite ways in different bacteria (Rosales-Reyes et al., 2012; Li et al., 2013). Interestingly, *SuhB* differential expression was also observed in two *R. solanacearum* strains (Meng et al., 2015). The function of Rhs (Rearrangement Hot Spot) proteins is ill-defined but they are considered to promote recombination (Lin et al., 1984). Interestingly, a member of the Rhs family was described to be induced during infection and associated with increased bacterial numbers and decreased survival in mice during pneumonia caused by *P. aeruginosa* (Kung et al., 2012). Finally, carbonic anhydrase catalyzes the inter-conversion between carbon dioxide and bicarbonate but is also required for growth of many animal pathogenic microorganisms (Capasso and Supuran, 2015). In addition, a role in disease establishment between potato and *Phytophthora infestans* was also reported (Restrepo et al., 2005), suggesting the possible implication of CAs during host colonization. These evidences suggest that *suhB, rhs*, and *RSUY_RS16085* encode putative virulence factors shared between gram-negative bacterial pathogens that infect animals and plants.

The assimilatory sulfate reduction pathway (c*ysD, cysN*, and *cysI*) and the phosphate mobilization (*pstB* and *pstS1*) were also induced during root colonization (Table 3). *cysD* and *cysN*, encode an ATP sulfurylase that produces APS, which can be in turn reduced to PAPS to ultimately synthesize cysteine by *cysI*. A study carried out in a closely related plant pathogenic bacterium, *Xanthomonas oryzae* pv. *Oryzae*, was demonstrated that mutation of either *raxP* or *raxQ* (homologs of *cysD* and *cysN*) impaired production of APS and PAPS and were required for the correct activity of the avirulence protein AvrXa21 (Shen et al., 2002). Further, several studies demonstrated that mutations on the pst system, responsible for phosphate uptake, affected virulence in diverse animal pathogenic bacteria (Rao et al., 2004; Lamarche et al., 2005, 2008). Altogether, these studies suggest that both systems might be regulators of bacterial pathogenicity, which could also be conserved in plant pathogens.

Finally, the *rtcA* and its regulator *rtcR* are down-regulated *in planta* (Table 3). The *rtc* system is involved in the regulation of the RNA repair system for ribosome homeostasis through the activation of *rtcR* by different agents and genetic lesions which in turn activates the *rtcAB* genes (Das and Shuman, 2013). The *rtc* system was also involved in the functioning of chemotaxis and motility in *Escherichia coli* (Engl et al., 2016), as mutations in either *rtcA* or *rtcB* increased motility. Since *rtc* acts a repressor of motility, its down-regulation in *S. commersonii* colonization could influence bacterial motility, a key virulence determinant.

## Author contributions

MP performed experiments, analyzed data and wrote the manuscript; RG analyzed data; PZ performed experiments; NC designed the research and wrote the manuscript; AM designed experiments and analyzed data; JS designed the research, analyzed data and wrote the manuscript; MV designed the research, performed experiments, analyzed data, and wrote the manuscript.

## Funding

This work was funded by projects AGL2013-46898-R, AGL2016-78002-R, and RyC 2014-16158 from the Spanish Ministry of Economy and Competitiveness. We also acknowledge financial support from the “Severo Ochoa Program for Centres of Excellence in R&D” 2016-2019 (SEV-2015-0533) and the CERCA Program of the Catalan Government (Generalitat de Catalunya) and from COST Action SUSTAIN (FA1208) from the European Union. APM is funded by the Chinese Academy of Sciences and the Chinese 1000 Talents Program. MP holds an APIF doctoral fellowship from Universitat de Barcelona and received a travel fellowship allowed by Fundació Montcelimar and Universitat de Barcelona to carry out a short stay in JCS's lab. RGS holds a doctoral fellowship; grant 2012/15197-1, São Paulo Research Foundation (FAPESP) and JCS has a CNPq research fellowship.

### Conflict of interest statement

The authors declare that the research was conducted in the absence of any commercial or financial relationships that could be construed as a potential conflict of interest.

## References

[B1] Ailloud F. Lowe T. M. Robene I. Cruveiller S. Allen C. Prior P. (2016). In planta comparative transcriptomics of host-adapted strains of Ralstonia solanacearum. PeerJ. 4:e1549. 10.7717/peerj.1549 26788428 PMC4715432

[B2] Anders S. Pyl P. T. Huber W. (2015). HTSeq–a Python framework to work with high-throughput sequencing data. Bioinformatics 31, 166–169. 10.1093/bioinformatics/btu638 25260700 PMC4287950

[B3] Bocsanczy A. M. Achenbach U. C. Mangravita-Novo A. Chow M. Norman D. J. (2014). Proteomic comparison o*f Ralstonia solanacearum* strains reveals temperature dependent virulence factors. BMC Genomics 15:280. 10.1186/1471-2164-15-280 24725348 PMC4023598

[B4] Boucher C. A. Van Gijsegem F. Barberis P. A. Arlat M. Zischek C. (1987). *Pseudomonas solanacearum* genes controlling both pathogenicity on tomato and hypersensitivity on tobacco are clustered. J. Bacteriol. 169, 5626–5632. 10.1128/jb.169.12.5626-5632.1987 2824440 PMC214013

[B5] Brown D. G. Allen C. (2004). *Ralstonia solanacearum* genes induced during growth in tomato: an inside view of bacterial wilt. Mol. Microbiol. 53, 1641–1660. 10.1111/j.1365-2958.2004.04237.x 15341645

[B6] Buttner D. (2016). Behind the lines-actions of bacterial type III effector proteins in plant cells. FEMS Microbiol. Rev. 40, 894–937. 10.1093/femsre/fuw026 28201715 PMC5091034

[B7] Capasso C. Supuran C. T. (2015). Bacterial, fungal and protozoan carbonic anhydrases as drug targets. Expert Opin. Ther. Targets 19, 1689–1704. 10.1517/14728222.2015.1067685 26235676

[B8] Capy P. Gasperi G. Biémont C. Bazin C. (2000). Stress and transposable elements: co-evolution or useful parasites? Heredity (Edinb). 85, 101–106. 10.1046/j.1365-2540.2000.00751.x 11012710

[B9] Casadevall A. Pirofski L. (2001). Host-pathogen interactions: the attributes of virulence. J. Infect. Dis. 184, 337–344. 10.1086/322044 11443560

[B10] Casado C. Llagostera M. Barbe J. (1991). Expression of nrdA and nrdB genes of *Escherichia coli* is decreased under anaerobiosis. FEMS Microbiol. Lett. 67, 153–157. 10.1111/j.1574-6968.1991.tb04432.x-i1 1778429

[B11] Colburn-Clifford J. Allen C. (2010). A cbb(3)-type cytochrome C oxidase contributes to *Ralstonia solanacearum* R3bv2 growth in microaerobic environments and to bacterial wilt disease development in tomato. Mol. Plant Microbe Interact. 23, 1042–1052. 10.1094/MPMI-23-8-1042 20615115

[B12] Coll N. S. Valls M. (2013). Current knowledge on the *Ralstonia solanacearum* type III secretion system. Microb. Biotechnol. 6, 614–620. 10.1111/1751-7915.12056 23617636 PMC3815929

[B13] Contreras-Moreira B. Vinuesa P. (2013). GET_HOMOLOGUES, a versatile software package for scalable and robust microbial pangenome analysis. Appl. Environ. Microbiol. 79, 7696–7701. 10.1128/aem.02411-13 24096415 PMC3837814

[B14] Cruz A. P. Ferreira V. Pianzzola M. J. Siri M. I. Coll N. S. Valls M. (2014). A novel, sensitive method to evaluate potato germplasm for bacterial wilt resistance using a luminescent *Ralstonia solanacearum* reporter strain. Mol. Plant Microbe Interact. 27, 277–285. 10.1094/MPMI-10-13-0303-FI 24283938

[B15] Cunnac S. Occhialini A. Barberis P. Boucher C. Genin S. (2004). Inventory and functional analysis of the large Hrp regulon in *Ralstonia solanacearum*: identification of novel effector proteins translocated to plant host cells through the type III secretion system. Mol. Microbiol. 53, 115–128. 10.1111/j.1365-2958.2004.04118.x 15225308

[B16] Dalsing B. L. Allen C. (2014). Nitrate assimilation contributes to *Ralstonia solanacearum* root attachment, stem colonization, and virulence. J. Bacteriol. 196, 949–960. 10.1128/JB.01378-13 24363343 PMC3957700

[B17] Dalsing B. L. Truchon A. N. Gonzalez-Orta E. T. Milling A. S. Allen C. (2015). *Ralstonia solanacearum* uses inorganic nitrogen metabolism for virulence, ATP production, and detoxification in the oxygen-limited host xylem environment. MBio 6:e02471. 10.1128/mBio.02471-14 25784703 PMC4453514

[B18] Das U. Shuman S. (2013). 2'-Phosphate cyclase activity of RtcA: a potential rationale for the operon organization of RtcA with an RNA repair ligase RtcB in *Escherichia coli* and other bacterial taxa. RNA 19, 1355–1362. 10.1261/rna.039917.113 23945037 PMC3854526

[B19] Delaspre F. Nieto Penalver C. G. Saurel O. Kiefer P. Gras E. Milon A.. (2007). The *Ralstonia solanacearum* pathogenicity regulator HrpB induces 3-hydroxy-oxindole synthesis. Proc. Natl. Acad. Sci. U.S.A. 104, 15870–15875. 10.1073/pnas.0700782104 17890323 PMC2000445

[B20] Engl C. Schaefer J. Kotta-Loizou I. Buck M. (2016). Cellular and molecular phenotypes depending upon the RNA repair system RtcAB of *Escherichia coli*. Nucleic Acids Res. 44, 9933–9941. 10.1093/nar/gkw628 27402162 PMC5175333

[B21] Flavier A. B. Ganova-Raeva L. M. Schell M. A. Denny T. P. (1997). Hierarchical autoinduction in *Ralstonia solanacearum*: control of Acyl-homoserine lactone production by a novel autoregulatory system responsive to 3-hydroxypalmitic acid methyl ester. J. Bacteriol. 179, 7089–7097. 10.1128/jb.179.22.7089-7097.1997 9371457 PMC179651

[B22] Flores-Cruz Z. Allen C. (2009). *Ralstonia solanacearum* encounters an oxidative environment during tomato infection. Mol. Plant Microbe Interact. 22, 773–782. 10.1094/MPMI-22-7-0773 19522559

[B23] Garriga X. Eliasson R. Torrents E. Barbé J. Gibert I. Reichard P. (1996). nrdD and nrdG genes are essential for strict anaerobic growth of *Escherichia coli*. Biochem. Biophys. Res. Commun. 229, 189–192. 10.1006/bbrc.1996.1778 8954104

[B24] Guarischi-Sousa R. Puigvert M. Coll N. S. Siri M. I. Pianzzola M. J. Valls M.. (2016). Complete genome sequence of the potato pathogen *Ralstonia solanacearum* UY031. Stand. Genomic Sci. 11, 7. 10.1186/s40793-016-0131-4 26779304 PMC4714475

[B25] Hamada M. Toyofuku M. Miyano T. Nomura N. (2014). cbb3-type cytochrome c oxidases, aerobic respiratory enzymes, impact the anaerobic life of *Pseudomonas aeruginosa* PAO1. J. Bacteriol. 196, 3881–3889. 10.1128/jb.01978-14 25182494 PMC4248832

[B26] Hayward A. C. (1991). Biology and epidemiology of bacterial wilt caused by *Pseudomonas solanacearum*. Annu. Rev. Phytopathol. 29, 65–87. 10.1146/annurev.py.29.090191.000433 18479193

[B27] Huang J. Carney B. F. Denny T. P. Weissinger A. K. Schell M. A. (1995). A complex network regulates expression of eps and other virulence genes of *Pseudomonas solanacearum*. J. Bacteriol. 177, 1259–1267. 10.1128/jb.177.5.1259-1267.1995 7868600 PMC176732

[B28] Jacobs J. M. Babujee L. Meng F. Milling A. Allen C. (2012). The in planta transcriptome of *Ralstonia solanacearum*: conserved physiological and virulence strategies during bacterial wilt of tomato. MBio 3:e00114–12. 10.1128/mBio.00114-12 22807564 PMC3413399

[B29] Jaeger C. H. III. Lindow S. E. Miller W. Clark E. Firestone M. K. (1999). Mapping of sugar and amino acid availability in soil around roots with bacterial sensors of sucrose and tryptophan. Appl. Environ. Microbiol. 65, 2685–2690. 10347061 10.1128/aem.65.6.2685-2690.1999 PMC91396

[B30] Kanda A. Yasukohchi M. Ohnishi K. Kiba A. Okuno T. Hikichi Y. (2003). Ectopic expression of *Ralstonia solanacearum* effector protein PopA early in invasion results in loss of virulence. Mol. Plant Microbe Interact. 16, 447–455. 10.1094/MPMI.2003.16.5.447 12744516

[B31] Kang Y. Liu H. Genin S. Schell M. A. Denny T. P. (2002). *Ralstonia solanacearum* requires type 4 pili to adhere to multiple surfaces and for natural transformation and virulence. Mol. Microbiol. 46, 427–437. 10.1046/j.1365-2958.2002.03187.x 12406219

[B32] Keen N. T. Tamaki S. Kobayashi D. Gerhold D. Stayton M. Shen H. (1990). Bacteria expressing avirulence Gene D produce a specific elicitor of the soybean hypersensitive reaction. Mol. Plant Microbe Interact. 3, 112–121. 10.1094/MPMI-3-112

[B33] Kim-Lee H. Moon J. S. Hong Y. J. Kim M. S. Cho H. M. (2005). Bacterial wilt resistance in the progenies of the fusion hybrids between haploid of potato and *Solanum commersonii*. Am. J. Potato Res. 82, 129–137. 10.1007/BF02853650

[B34] Kredich N. M. (1992). The molecular basis for positive regulation of cys promoters in *Salmonella typhimurium* and *Escherichia coli*. Mol. Microbiol. 6, 2747–2753. 10.1111/j.1365-2958.1992.tb01453.x 1435253

[B35] Kung V. L. Khare S. Stehlik C. Bacon E. M. Hughes A. J. Hauser A. R. (2012). An rhs gene of *Pseudomonas aeruginosa* encodes a virulence protein that activates the inflammasome. Proc. Natl. Acad. Sci. U.S.A. 109, 1275–1280. 10.1073/pnas.1109285109 22232685 PMC3268321

[B36] La M. V. Raoult D. Renesto P. (2008). Regulation of whole bacterial pathogen transcription within infected hosts. FEMS Microbiol. Rev. 32, 440–460. 10.1111/j.1574-6976.2008.00103.x 18266740

[B37] Lamarche M. G. Dozois C. M. Daigle F. Caza M. Curtiss R. III. Dubreuil J. D.. (2005). Inactivation of the pst system reduces the virulence of an avian pathogenic *Escherichia coli* O78 strain. Infect. Immun. 73, 4138–4145. 10.1128/iai.73.7.4138-4145.2005 15972503 PMC1168596

[B38] Lamarche M. G. Wanner B. L. Crepin S. Harel J. (2008). The phosphate regulon and bacterial virulence: a regulatory network connecting phosphate homeostasis and pathogenesis. FEMS Microbiol. Rev. 32 461–473. 10.1111/j.1574-6976.2008.00101.x 18248418

[B39] Langmead B. Salzberg S. L. (2012). Fast gapped-read alignment with Bowtie 2. Nat. Methods 9, 357–359. 10.1038/nmeth.1923 22388286 PMC3322381

[B40] Leimbach A. (2016). Bac-Genomics-Scripts: Bovine E. coli Mastitis Comparative Genomics Edition [Data set]. Zenodo 10.5281/zenodo.215824

[B41] Li K. Xu C. Jin Y. Sun Z. Liu C. Shi J.. (2013). SuhB is a regulator of multiple virulence genes and essential for pathogenesis of *Pseudomonas aeruginosa*. MBio 4, e00419–e00413. 10.1128/mbio.00419-13 24169572 PMC3809559

[B42] Lin R. J. Capage M. Hill C. W. (1984). A repetitive DNA sequence, rhs, responsible for duplications within the *Escherichia coli* K-12 chromosome. J. Mol. Biol. 177, 1–18. 10.1016/0022-2836(84)90054-8 6086936

[B43] Liu H. Kang Y. Genin S. Schell M. A. Denny T. P. (2001). Twitching motility of *Ralstonia solanacearum* requires a type IV pilus system. Microbiology (Reading,. Engl). 147, 3215–3229. 10.1099/00221287-147-12-3215 11739754

[B44] Lonjon F. Turner M. Henry C. Rengel D. Lohou D. van de Kerkhove Q.. (2016). Comparative secretome analysis of *Ralstonia solanacearum* Type 3 secretion-associated mutants reveals a fine control of effector delivery, essential for bacterial pathogenicity. Mol. Cell. Proteomics. 15, 598–613. 10.1074/mcp.M115.051078 26637540 PMC4739675

[B45] Love M. I. Huber W. Anders S. (2014). Moderated estimation of fold change and dispersion for RNA-seq data with DESeq2. Genome Biol. 15:550. 10.1186/s13059-014-0550-8 25516281 PMC4302049

[B46] Lundgren B. R. Connolly M. P. Choudhary P. Brookins-Little T. S. Chatterjee S. Raina R.. (2015). Defining the metabolic functions and roles in virulence of the rpoN1 and rpoN2 Genes in *Ralstonia solanacearum* GMI1000. PLoS ONE 10:e0144852. 10.1371/journal.pone.0144852 26659655 PMC4676750

[B47] Meng F. (2013). The virulence factors of the bacterial wilt pathogen *Ralstonia solanacearum*. J. Plant Pathol. Microbiol. 4:168. 10.4172/2157-7471.1000168

[B48] Meng F. Babujee L. Jacobs J. M. Allen C. (2015). Comparative transcriptome analysis reveals cool virulence factors of *Ralstonia solanacearum* Race 3 Biovar 2. PLoS ONE 10:e0139090. 10.1371/journal.pone.0139090 26445498 PMC4596706

[B49] Milewski S. (2002). Glucosamine-6-phosphate synthase–the multi-facets enzyme. Biochim. Biophys. Acta 1597, 173–192. 10.1016/S0167-4838(02)00318-7 12044898

[B50] Monteiro F. Genin S. van Dijk I. Valls M. (2012). A luminescent reporter evidences active expression of *Ralstonia solanacearum* type III secretion system genes throughout plant infection. Microbiology (Reading,. Engl). 158, 2107–2116. 10.1099/mic.0.058610-0 22609750

[B51] Mori Y. Inoue K. Ikeda K. Nakayashiki H. Higashimoto C. Ohnishi K.. (2016). The vascular plant-pathogenic bacterium *Ralstonia solanacearum* produces biofilms required for its virulence on the surfaces of tomato cells adjacent to intercellular spaces. Mol. Plant Pathol. 17, 890–902. 10.1111/mpp.12335 26609568 PMC6638453

[B52] Mucyn T. S. Yourstone S. Lind A. L. Biswas S. Nishimura M. T. Baltrus D. A.. (2014). Variable suites of non-effector genes are co-regulated in the type III secretion virulence regulon across the Pseudomonas syringae phylogeny. PLoS Pathog. 10:e1003807. 10.1371/journal.ppat.1003807 24391493 PMC3879358

[B53] Occhialini A. Cunnac S. Reymond N. Genin S. Boucher C. (2005). Genome-wide analysis of gene expression in *Ralstonia solanacearum* reveals that the hrpB gene acts as a regulatory switch controlling multiple virulence pathways. Mol. Plant Microbe Interact. 18, 938–949. 10.1094/MPMI-18-0938 16167764

[B54] Peeters N. Guidot A. Vailleau F. Valls M. (2013). Ralstonia solanacearum, a widespread bacterial plant pathogen in the post-genomic era. Mol. Plant Pathol. 15, 651–662. 10.1111/mpp.12038 PMC6638647 23718203

[B55] Pegg G. F. (1985). Life in a black hole: the microenvironment of the vascular pathogen. Trans. Brit. Mycol. Soc. 85, 1–20. 10.1016/S0007-1528(85)80043-2

[B56] Peyraud R. Cottret L. Marmiesse L. Gouzy J. Genin S. (2016). A resource allocation trade-off between virulence and proliferation drives metabolic versatility in the plant pathogen *Ralstonia solanacearum*. PLoS Pathog. 12:e1005939. 10.1371/journal.ppat.1005939 27732672 PMC5061431

[B57] Plener L. Boistard P. Gonzalez A. Boucher C. Genin S. (2012). Metabolic adaptation of *Ralstonia solanacearum* during plant infection: a methionine biosynthesis case study. PLoS ONE 7:e36877. 10.1371/journal.pone.0036877 22615832 PMC3353975

[B58] Popa C. Coll N. S. Valls M. Sessa G. (2016a). Yeast as a heterologous model system to uncover type III effector function. PLoS Pathog. 12:e1005360. 10.1371/journal.ppat.1005360 26914889 PMC4767418

[B59] Popa C. Li L. Gil S. Tatjer L. Hashii K. Tabuchi M.. (2016b). The effector AWR5 from the plant pathogen *Ralstonia solanacearum* is an inhibitor of the TOR signalling pathway. Sci. Rep. 6:27058. 10.1038/srep27058 27257085 PMC4891724

[B60] Priou S. (2004). Integrated Management of Bacterial Wilt and Soil-Borne Diseases of Potato in Farmer Communities of the Inter-Andean Valleys of Peru and Bolivia. Final Technical Report DFID-funded project CRF 7862(C), CIP, Lima.

[B61] Rao P. S. Yamada Y. Tan Y. P. Leung K. Y. (2004). Use of proteomics to identify novel virulence determinants that are required for *Edwardsiella tarda* pathogenesis. Mol. Microbiol. 53, 573–586. 10.1111/j.1365-2958.2004.04123.x 15228535

[B62] Ray S. K. Kumar R. Peeters N. Boucher C. Genin S. (2015). rpoN1, but not rpoN2, is required for twitching motility, natural competence, growth on nitrate, and virulence of *Ralstonia solanacearum*. Front. Microbiol. 6:229. 10.3389/fmicb.2015.00229 25852679 PMC4371752

[B63] Restrepo S. Myers K. del Pozo O. Martin G. B. Hart A. L. Buell C. R.. (2005). Gene profiling of a compatible interaction between Phytophthora infestans and *Solanum tuberosum* suggests a role for carbonic anhydrase. Mol. Plant Microbe Interact. 18, 913–922. 10.1094/MPMI-18-0913 16167762

[B64] Robinson J. T. Thorvaldsdottir H. Winckler W. Guttman M. Lander E. S. Getz G.. (2011). Integrative genomics viewer. Nat. Biotechnol. 29, 24–26. 10.1038/nbt.1754 21221095 PMC3346182

[B65] Rocha E. R. Smith C. J. (1999). Role of the alkyl hydroperoxide reductase (ahpCF) Gene in oxidative stress defense of the obligate. J. Bacteriol. 181, 5701–5710. 10482511 10.1128/jb.181.18.5701-5710.1999 PMC94090

[B66] Roessner C. A. Scott A. I. (2006). Fine-tuning our knowledge of the anaerobic route to cobalamin (vitamin B12). J. Bacteriol. 188, 7331–7334. 10.1128/jb.00918-06 16936030 PMC1636268

[B67] Rosales-Reyes R. Saldias M. S. Aubert D. F. El-Halfawy O. M. Valvano M. A. (2012). The suhB gene of *Burkholderia cenocepacia* is required for protein secretion, biofilm formation, motility and polymyxin B resistance. Microbiology 158, 2315–2324. 10.1099/mic.0.060988-0 22767545

[B68] Schell M. A. (2000). Control of virulence and pathogenicity genes of *Ralstonia Solanacearum* by an elaborate sensory network. Annu. Rev. Phytopathol. 38, 263–292. 10.1146/annurev.phyto.38.1.263 11701844

[B69] Shen Y. Sharma P..da Silva F. G. Ronald P. (2002). The Xanthomonas oryzae pv. oryzae raxP and raxQ genes encode an ATP sulphurylase and adenosine-5 /c-phosphosulphate kinase that are required for AvrXa21avirulence activity. Mol. Microbiol. 44, 37–48. 10.1046/j.1365-2958.2002.02862.x 11967067

[B70] Shinohara R. Kanda A. Ohnishi K. Kiba A. Hikichi Y. (2005). Contribution of folate biosynthesis to *Ralstonia solanacearum* proliferation in intercellular spaces. Appl. Environ. Microbiol. 71, 417–422. 10.1128/AEM.71.1.417-422.2005 15640216 PMC544231

[B71] Singh N. Bubunenko M. Smith C. Abbott D. M. Stringer A. M. Shi R.. (2016). SuhB associates with nus factors to facilitate 30S ribosome biogenesis in *Escherichia coli*. MBio 7:e00114. 10.1128/mBio.00114-16 26980831 PMC4807359

[B72] Siri M. I. Galván G. A. Quirici L. Silvera E. Villanueva P. Ferreira F. (2009). Molecular marker diversity and bacterial wilt resistance in wild *Solanum commersonii* accessions from Uruguay. Euphytica 165, 371–382. 10.1007/s10681-008-9800-8

[B73] Siri M. I. Sanabria A. Boucher C. Pianzzola M. J. (2014). New type IV pili-related genes involved in early stages of *Ralstonia solanacearum* potato infection. Mol. Plant Microbe Interact. 27 712–724. 10.1094/MPMI-07-13-0210-R 24625029

[B74] Siri M. I. Sanabria A. Pianzzola M. J. (2011). Genetic diversity and aggressiveness of ralstonia solanacearumstrains causing bacterial wilt of potato in uruguay. Plant Dis. 95, 1292–1301. 10.1094/pdis-09-10-0626 30731700

[B75] Sole M. Popa C. Mith O. Sohn K. H. Jones J. D. Deslandes L.. (2012). The awr gene family encodes a novel class of *Ralstonia solanacearum* type III effectors displaying virulence and avirulence activities. Mol. Plant Microbe Interact. 25, 941–953. 10.1094/MPMI-12-11-0321 22414437

[B76] Stes E. Vandeputte O. M. El Jaziri M. Holsters M. Vereecke D. (2011). A successful bacterial coup d'etat: how *Rhodococcus fascians* redirects plant development. Annu. Rev. Phytopathol. 49, 69–86. 10.1146/annurev-phyto-072910-095217 21495844

[B77] Tans-Kersten J. Brown D. Allen C. (2004). Swimming motility, a virulence trait of *Ralstonia solanacearum*, is regulated by FlhDC and the plant host environment. Mol. Plant Microbe Interact. 17, 686–695. 10.1094/MPMI.2004.17.6.686 15195951

[B78] Thorvaldsdottir H. Robinson J. T. Mesirov J. P. (2013). Integrative Genomics Viewer (IGV): high-performance genomics data visualization and exploration. Brief. Bioinform. 14, 178–192. 10.1093/bib/bbs017 22517427 PMC3603213

[B79] Ullmann A. Danchin A. (1980). Role of cyclic AMP in regulatory mechanisms in bacteria. Trends Biochem. Sci. 5, 95–96. 10.1016/0968-0004(80)90257-1

[B80] Valls M. Genin S. Boucher C. (2006). Integrated regulation of the type III secretion system and other virulence determinants in *Ralstonia solanacearum*. PLoS Pathog. 2:e82. 10.1371/journal.ppat.0020082 16933989 PMC1557829

[B81] Wenk M. R. (2006). Lipidomics of host-pathogen interactions. FEBS Lett. 580, 5541–5551. 10.1016/j.febslet.2006.07.007 16859687

[B82] Yu X. Lund S. P. Scott R. A. Greenwald J. W. Records A. H. Nettleton D.. (2013). Transcriptional responses of Pseudomonas syringae to growth in epiphytic versus apoplastic leaf sites. Proc. Natl. Acad. Sci. U.S.A. 110, E425–E434. 10.1073/pnas.1221892110 23319638 PMC3562829

[B83] Zuluaga A. P. Puigvert M. Valls M. (2013). Novel plant inputs influencing *Ralstonia solanacearum* during infection. Front. Microbiol. 4:349. 10.3389/fmicb.2013.00349 24312090 PMC3834233

[B84] Zuluaga A. P. Sole M. Lu H. Gongora-Castillo E. Vaillancourt B. Coll N.. (2015). Transcriptome responses to *Ralstonia solanacearum* infection in the roots of the wild potato *Solanum commersonii*. BMC Genomics 16:246. 10.1186/s12864-015-1460-1 25880642 PMC4391584

