# Supplementary figures and images for: Transcriptomes of Ralstonia solanacearum during Root Colonization of Solanum commersonii
Source: Front Plant Sci. 2017 Mar 20;8:370. doi: 10.3389/fpls.2017.00370 (PMC5357869; doi:10.3389/fpls.2017.00370)

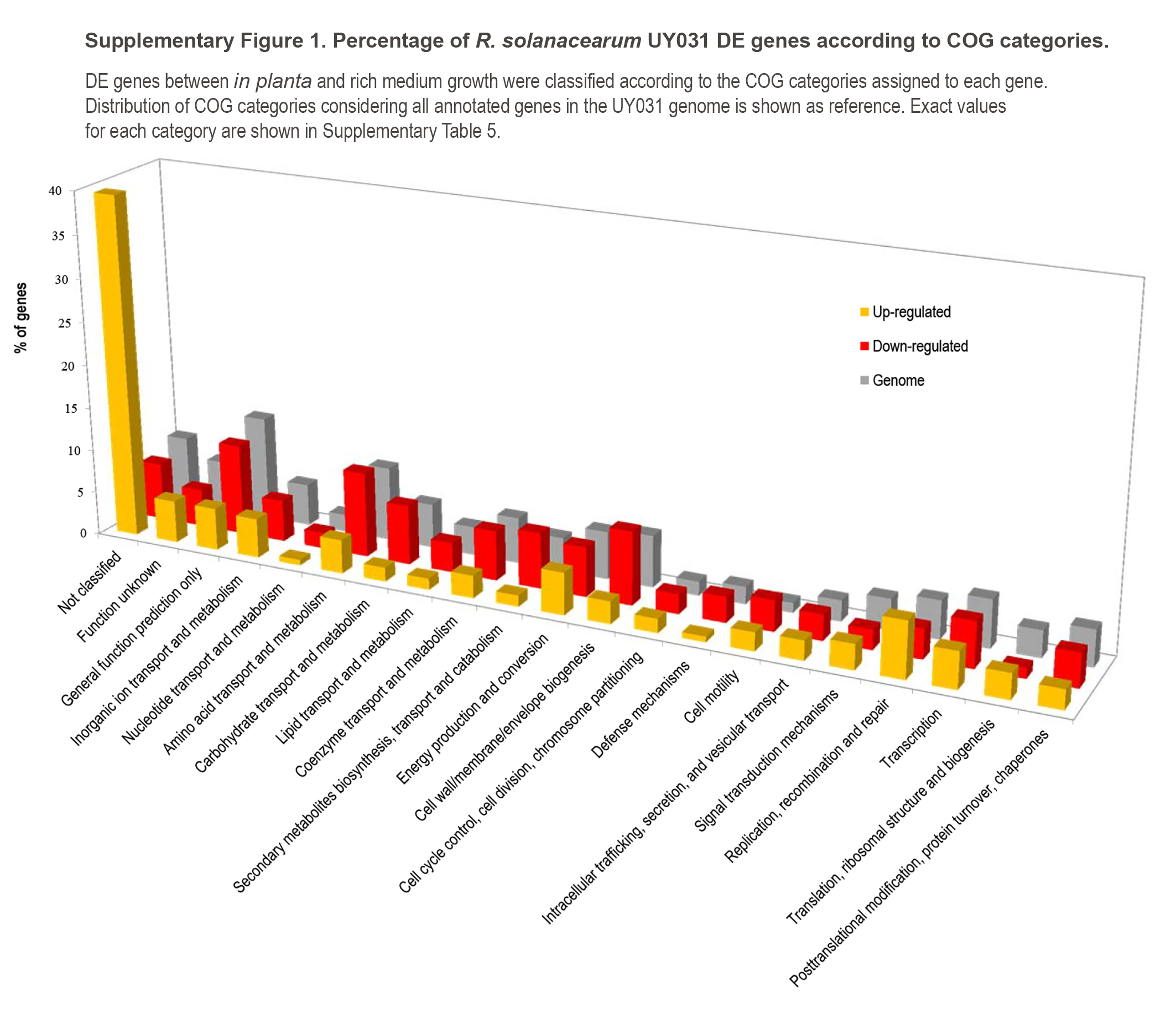

Supplement: Supplementary file 6 [file Image1.tif]
